# A Routine PET/CT Protocol with Streamlined Calculations for Assessing Cardiac Amyloidosis Using ^18^F-Florbetapir

**DOI:** 10.3389/fcvm.2015.00023

**Published:** 2015-05-08

**Authors:** Dustin R. Osborne, Shelley N. Acuff, Alan Stuckey, Jonathan S. Wall

**Affiliations:** ^1^Molecular Imaging and Translational Research Program, Department of Radiology, Graduate School of Medicine, University of Tennessee, Knoxville, TN, USA; ^2^Amyloid and Cancer Theranostics Program, Department of Medicine, Graduate School of Medicine, University of Tennessee, Knoxville, TN, USA

**Keywords:** cardiac, amyloidosis, amyloid, PET/CT, Florbetapir

## Abstract

**Introduction:**

Cardiac amyloidosis is a rare condition characterized by the deposition of well-structured protein fibrils, proteoglycans, and serum proteins as amyloid. Recent work has shown that it may be possible to use ^18^F-Florbetapir to image cardiac amyloidosis. Current methods for assessment include invasive biopsy techniques. This work enhances foundational work by Dorbala et al. by developing a routine imaging and analysis protocol using ^18^F-Florbetapir for cardiac amyloid assessment.

**Methods:**

Eleven patients, three healthy controls and eight myloid positive patients, were imaged using ^18^F-Florbetapir to assess cardiac amyloid burden. Four of the patients were also imaged using ^82^Rb-Chloride to evaluate possible ^18^F-Florbetapir retention because of reduced myocardial blood flow. Quantitative methods using modeling, SUVs and SUV ratios were used to define a new streamlined clinical imaging protocol that could be used routinely and provide patient stratification.

**Results:**

Quantitative analysis of ^18^F-Florbetapir cardiac amyloid data were compiled from a 20-min listmode protocol with data histogrammed into two static images at 0–5, 10–15, or 15–20 min. Data analysis indicated the use of SUVs or ratios of SUVs calculated from regions draw in the septal wall were adequate in identification of all healthy controls from amyloid positive patients in this small cohort. Additionally, we found that it may be possible to use this method to differentiate patients suffering from AL vs. TTR amyloid.

**Conclusion:**

This work builds on the seminal work by Dorbala et al. by describing a short ^18^F-Florbetapir imaging protocol that is suitable for routine clinical use and uses a simple method for quantitative analysis of cardiac amyloid disease.

## 1 Introduction

Cardiac amyloidosis is a rare condition characterized by the deposition of well-structured protein fibrils, proteoglycans, and serum proteins as amyloid (1). The most common forms result from the deposition of monoclonal immunoglobulin light chains (AL amyloidosis) or transthyretin, either wild type (wtATTR) or mutant (ATTR) protein (2). The majority of patients with AL and ATTR develop systemic amyloidosis involving all visceral organs, particularly the liver, spleen, kidneys, and heart in patients with AL, and peripheral nerves and heart in patients with ATTR. Cardiac amyloidosis described as early as 1936 (3), typically remains un-diagnosed until the deposits become extensive, primarily because patients have a long asymptomatic phase and present with common and non-specific symptoms that can range from decreased appetite to gastrointestinal problems (4). Patients with advanced cardiac involvement often present with symptoms of cardiomyopathy, such as shortness of breath (5). Therefore, the disease is often initially misdiagnosed as a restrictive cardiomyopathy, failure, e.g., associated with diabetes, without amyloid involvement (6). Misdiagnosis may result in improper patient management and a delay in appropriate patient care and intervention.

Prognosis for patients with amyloidosis and cardiac involvement is generally poor with median survival rates at diagnosis of 5–6 months for AL patients and 3–5 years for ATTR patients (7, 8). Improved therapeutic strategies for the prevention of AL and ATTR amyloid are being developed. Furthermore, novel immunotherapies for amyloid removal are in clinical trial and may provide new options for patients with amyloidosis (9). The definitive diagnosis of systemic amyloidosis generally requires a sub-cutaneous fat biopsy followed by Congo red staining of the material amyloid is visible as blue–green birefringent material when the slide is viewed microscopically under cross-polarized illumination. Cardiac involvement is often identified by changes in the serum levels of brain natriuretic peptide (10) as well as the presence of interventricular thickening seen following ultrasound and/or magnetic resonance (MR) imaging (11). Although useful, neither of these techniques are amyloid specific nor provide quantitative data on amyloid load. To this end, molecular imaging strategies for detecting cardiac amyloidosis have been applied but are not widely adopted outside of specialized amyloid clinics (12). Gamma scintigraphic imaging with ^99^*^m^*Tc-labeled 3,3-diphosphono-1,2 propanodicarboxylic acid (^99^*^m^*Tc-DPD) can provide accurate imaging of ATTR cardiac amyloid, but not cardiac AL or TTR amyloid deposits in organs other than the heart (13). ^99^*^m^*Tc-labeled aprotinin has been used to image cardiac amyloidosis in patients with AL and ATTR (14), but is not widely used since the withdrawal of bovine aprotinin from the market (15, 16). Anatomic imaging using echocardiography or magnetic resonance imaging can be effectively used to determine the extent of the amyloid involvement in the myocardium, but is non-specific for disease etiology (17, 18).

Recent studies have shown that cardiac amyloidosis can be detected using 11C-labeled Pittsburgh Compound B (11C-PIB); however, parametric imaging was required and amyloid in other visceral organs was not imaged (19). This work, although clearly showing potential benefits, is limited for general application by a number of compounding factors. More recently, small molecule radiotracers used for detecting A amyloid in the brains of patients with Alzheimers disease have been validated and approved by the US Food and Drug Administration (20).

Dorbala et al. have provided the foundational work assessing the potential role of ^18^F-Florbetapir (^18^F-AV45, Amyvid) in imaging cardiac amyloid patients (21). They show that using a 30–60 min scan time and parametric analysis, patients can be suitably diagnosed and staged. Our work builds upon these results through the development of a standardized imaging protocol of approximately 20 min and through the development of simplified analysis mechanisms to provide a quantitative assessment of cardiac amyloid in patients (22, 23). We also assess the role of cardiac perfusion in cardiac amyloid patients to determine whether ^18^F-Florbetapir imaging results could potentially be altered by perfusion phenomenon.

## 2 Materials and Methods

### 2.1 Patient population

Written informed consent for all patients was obtained under two protocols approved by the University of Tennessee Institutional Review Board (#3420 and #3841). Eight subjects, three with ATTR and five with AL amyloid, were recruited for this study. Disease was confirmed in accordance with criteria established for the diagnosis of cardiac amyloidosis (24) using endomyocardial biopsy or cardiac magnetic resonance imaging (MRI) with tissue biopsy. Three healthy, control subjects (HC) were recruited and imaged by using MRI to verify the absence of cardiac amyloidosis. All patients were imaged using ^18^F-Florbetapir. Three of the eight cardiac amyloid patients and one HC were imaged with rubidium chloride (82Rb-chloride) to assess myocardial perfusion. Additional details regarding clinical characteristics of the patient recruited into this study can be found in Table 1.

**Table 1:** **Patient data for amyloid positive and healthy controls**.

Patient type	Treatment	IVS	Rb PET/CT	Ejection fraction	Biopsy or cMRI	BNP
TTR	Dolobid	1.8	N/A	Echo: <50%	Heart: TTR+	554
				cMRI: 55%	MRI: +	
TTR	SCT 2002	1.9	N/A	Echo: 55%	Fat aspirate: apo A4	706
					cMRI: +	
Cntrl	N/A	1.1	N/A	Echo: <60%	N/A	N/A
TTR	None	1.7	N/A	Echo: <65%	Fat aspirate: TTR+	38
					cMRI: +	
AL	Velcade	1.8	Normal	Echo: <65%	Omentum: +	72
				Rb: 53%	cMRI: +	
AL	None	1.4	N/A	N/A	Fat aspirate: 1+	508
TTR	Dolobid	2.2	N/A	Echo: 45%	Heart: TTR+	515
Cntrl	N/A	1.1	N/A	Echo: 60%	N/A	N/A
Cntrl	N/A	0.9	Normal	Echo: <60%	N/A	N/A
				Rb: 73%		
AL	Cyclosporine	1.3	Normal	Echo: <60%	Kidney: 3+	501
	Velcade			Rb: 60%	cMRI: +	
	Dexamethasone					
AL	Velcade	0.7	Normal	Echo: 67%	Kidney: +	563
	Dexamethasone			Rb: 59%	cMRI: +	

### 2.2 ^18^F-Florbetapir PET/CT imaging

Patients were imaged on either a Biograph 6 TruePoint PET/CT or a Biograph 64 mCT Flow (Siemens Medical Solutions USA, Inc.). Each subject was placed on the imaging table and injected with 10 mCi of ^18^F-Florbetapir 10–15 s after the start of listmode data acquisition. Image data were acquired at a single-bed position with the patient heart in the center of the field of view. PET data were acquired for 30 min based on findings from previous work (22). A low-dose CT (120 kVp, with variable mAs) was acquired for rough anatomical localization and attenuation and scatter correction. CT data were reconstructed using a 512 × 512 image matrix with 1.37 mm isotropic voxels and 5 mm slice thickness.

PET data were histogrammed into 35 frames for dynamic analysis (10 frames of 10 s each, 10 frames of 30 s each, 10 frames of 60 s each, 3 frames of 255 s each) as well as into single static sinograms for reconstruction. PET data were reconstructed using OSEM iterative reconstruction algorithms to reconstruct a 200 × 200 image matrix with 2.67 mm isotropic voxel and a 2 mm slice thickness. All PET data were reconstructed with point spread function resolution recovery (HD•PET, Siemens Medical Solutions USA, Inc.) applied.

A complete outline of the clinical protocol is provided in the Section 3.

### 2.3 ^18^F-Florbetapir image analysis

Regions of interest (ROIs) were drawn over the left and right ventricular myocardium, blood pool, and portions of the liver. Details regarding these regions are described below. Similar to Dorbala et al. standard uptake values (SUVs), descriptive statistics, and time activity curves (TACs) were generated for each ROI. Left and right ventricle uptake was compared for each population to assess any differences. TACs were analyzed for each ROI and data were fit to multi-exponentials for basic kinetic assessment of uptake and washout rates. Quantitative values were compared between control and disease populations.

Data were assessed in four ways:

- First: SUVs were calculated for ROIs drawn in the septal wall (1 cc volume) and near the dome of the liver (3 cc volume) from data summed over 0–3, 10–15, and 15–20 min. SUV data for patients and HC were compared.
- Second: static images were created by summing data from 0–3 min (3), 10–15 min (10), and 15–20 min (15).
- Third: ROIs drawn on the septal wall were used to calculate mean, maximum, and minimum SUVs for each set of summed data. SUV ratios were then calculated for (0–3 min)/(10–15 min) (3:10) and (0–3 min)/(15–20 min) (3:15) to assess the potential of a simple ratiometric method for determining the presence of cardiac amyloidosis.
- Fourth: TAC data were used to determine the greatest range between SUV maxima and minima within the 20 min imaging time frame. Analysis of TAC data was used to assess the maximum SUV within the first 3 min of imaging as well as minimum values between 10–15 and 15–20 min. Ratios of maximum to minimum SUV for each range of times were then calculated.

Left and right ventricular variation of ^18^F-Florbetapir uptake was assessed using both intra- and inter-patient observations. Both of these were performed to assess the data for potential differences in ventricular uptake in each individual and between confirmed cardiac amyloid patients and healthy controls.

### 2.4 Rubidium PET/CT imaging protocol

Patients were injected with approximately 5 mCi of ^82^Rb-Chloride using a Cardiogen-82 rubidium generator and injection system (Bracco, Prairie, MN, USA). Image data were acquired for 7 min with dynamic processing into 16 frames per routine clinically used standards (25). As this perfusion study was only to measure impact on resting ^18^F-Florbetapir uptake, patients were only asked to perform the test at a resting heart rate and were not subjected to undue pharmacological stress testing. This would have only been appropriate if we were attempting to determine changes in heart function between stress and rest.

All dynamic PET data were corrected for attenuation and scatter using a low-dose CT protocol of 120 kVp and continuously modulated mAs. CT data were not used for analysis of the perfusion data. Corrected data were reconstructed using an iterative OSEM algorithm to create a 200 × 200 matrix with 2.05 mm isotropic voxels and 5 mm slice thickness.

### 2.5 ^82^Rubidium image analysis

Adding to the current body of cardiac amyloid work using ^82^Rb-Chloride compared to ^18^F-Florbetapir, analysis of myocardial blood flow was performed using the FlowQuant software package (Ottawa Heart Institute, Ottawa, ON, Canada). This software is widely considered as a gold standard for cardiac image analysis and has been thoroughly validated against common perfusion analysis software used clinically (26, 27). The automated algorithms and workflows in this software were used for reorientation and segmentation of the heart. Visual confirmation was used throughout the process to validate the placement of regions of interest used for modeling or make adjustments with the selection. Partial volume corrections were applied to image derived input functions drawn in the left ventricle to improve quantitative accuracy in these small regions.

### 2.6 Statistical analysis

A comparison of myocardial blood flow results from ^82^Rb-chloride modeling analysis and quantitative analysis from ^18^F-Florbetapir was performed to determine any potential correlation between ^18^F-Florbetapir uptake and decreased heart function as a result of amyloid disease. Pearson correlation analysis was used to assess the correlation between myocardial blood flow and cardiac amyloid burden with *p* < 0.05 considered significant for all statistical tests in this work. Confidence intervals (CI) of 95% were calculated and discriminant function analysis (DFA) was used to assess the models ability to stratify patient populations and determine the fit of the model to the data. *Post hoc* power analysis was performed to assess statistical power where applicable.

In addition to our correlation of ^82^Rb-Chloride perfusion to ^18^F-Florbetapir uptake, we assessed any potential correlation between left ventricular mass and ^18^F-Florbetapir uptake. Left ventricular mass was calculated using the formulation by Devereux et al. (28) and correlation analysis performed between LV mass and ^18^F-Florbetapir maximum and mean activity concentrations in the myocardial regions of interest. Statistical correlation analysis was performed by calculating Spearman correlation coefficients.

## 3 Results

### 3.1 ^18^F-Florbetapir cardiac analysis

**Figure 1:**
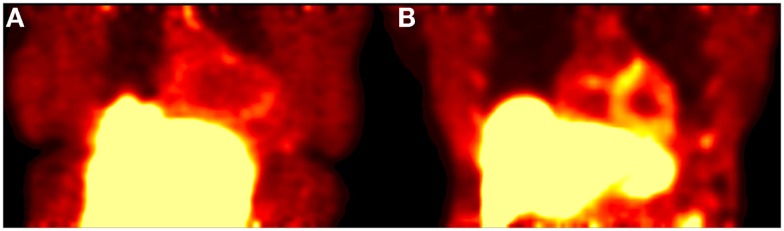
**Shows a 20-min acquisition of healthy (A) and amyloid positive (B) patients**. Both images were acquired at 1 h post injection.

Visual assessment of static ^18^F-Florbetapir images showed differences between HC and patients with cardiac amyloidosis in the form of increased uptake in regions of the heart associated with amyloid burden (Figure 1). Accurate visual assessment was highly dependent upon the imaging time frames used.

**Figure 2:**
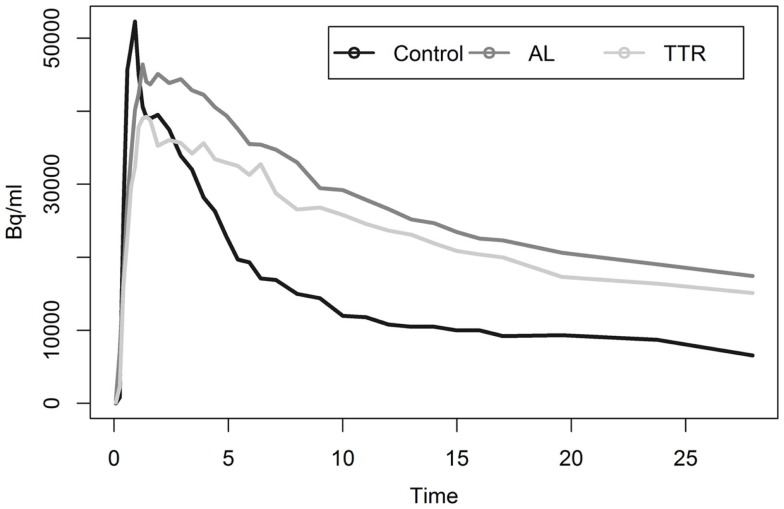
**Shows average myocardial TACs for healthy and amyloid positive populations**.

Analysis of myocardial TACs revealed significant differences (*p* < 0.05) in estimates of washout rates for patients with cardiac amyloid and HCs (Figure 2). Multi-exponential fits indicated an increased uptake rate for HC as compared to patients with calculated rates of 0.392 and 0.075 min^−1^, respectively.

Most noticeable was the difference in uptake rates between 0 and 20 min post injection (p.i.), where the average rate of change of SUV for HC was 5.4 SUV/min as compared to 0.28 SUV/min for amyloid patients [t(9) = −5.96, *p* < 0.01]. SUVs calculated using ROIs drawn on summed 5 min frames starting at 10 or 15 min p.i. showed statistically significant (*p* < 0.05) difference between the mean SUV for patients and HC (Table 2). SUV measurements at 10 min p.i. provided the largest difference between amyloid patients and HC (Table 3). None of the HC had an SUV >2 at 10 or 15 min p.i. Assessment of correlation between activity concentration and left ventricular mass yielded no significant relationships with *p* > 0.05 for all calculations of Pearson and Spearman correlation coefficients. *Post hoc* power analysis indicated a power of >90%.

**Table 2:** **SUV measurements at different time points**.

Subjects	3 min SUVAvg	10 min SUVAvg	15 min SUVAvg
Healthy controls	8.7 ± 0.6	1.7 ± 0.3	1.4 ± 0.2
Amyloid	8.4 ± 4.4	6.1 ± 1.6	4.7 ± 1.7

**Table 3:** **SUV ratio values**.

Subject	3:10 min ratio	3:15 min ratio
Healthy controls	5.12 ± 0.99	6.2 ± 0.95
Amyloid	1.4 ± 0.6	2.3 ± 1.4

No differences in left and right ventricular uptake were observed between in any single patient or when comparing healthy controls and those with confirmed cardiac amyloid disease (*p* < 0.05). No significant uptake was found in the ventricles with SUVs of no more than three for any patient in our study after peak values were reached within the first 3 min of data acquisition.

### 3.2 ^18^F-Florbetapir analysis: Discriminant function analysis

Discriminant function analysis verified that use of SUV alone at 10 and 15 min time points created a statistically significant model for patient stratification even in the small population used in this study. Use of 95% CI classification methods did not work as a useful model for the 15-min time point but was an accurate classification method for the 10-min time point data. Differences in ratios calculated for healthy and disease populations were statistically significant (*p* < 0.005) for all ratio methods tested as shown in Table 4.

**Table 4:** ***t*-Test results for SUV ratios**.

	*t*-Score	Sig. (2-tailed)	Mean difference	SE difference	95% Confidence interval of the difference	
					Lower	Upper
***t*-Test for equality of means**
3:10 TAC	9.15	0	7.03	0.77	5.26	8.8
3:10 Mean	7.25	0	3.7	0.51	2.52	4.87
3:15 TAC	6.34	0	7.66	1.21	4.87	10.44
3:15 Mean	5.84	0	4.2	0.72	2.54	5.85

Discriminant function analysis verified that ratios of SUVs at 3:10 and 3:15 min correctly classified all of the patient population tested (*n* = 10) and was a statistically significant (*p* < 0.01) model. The 3:10 min ratio resulted in a greater canonical correlation (93.2%) as compared to the 3:15 ratio, which resulted in a 90% correlation indicating that either time point ratio of static SUVs would be a suitable model to classify the data. Lower threshold limits for SUV mean ratios for 3:10 and 3:15 ratios were 4.7 and 5.3, respectively.

### 3.3 ^18^F-Florbetapir analysis: Ratio methods

Ratio methods derived from seeking maximum and minimum values within the regions of interest of a given time frame yielded similar results able to classify samples with >90% accuracy with statistical significance (*p* < 0.001). Models using these maximized ratios improved canonical correlation of the model to the data compared to using only the mean SUVs from static images. Maximized ratios of 3:10 and 3:15 min resulted in canonical correlations of 95.5% (2.4% increase) and 91.3% (1.4% increase), respectively. *Post hoc* power analysis indicated a power of >90%.

**Figure 3:**
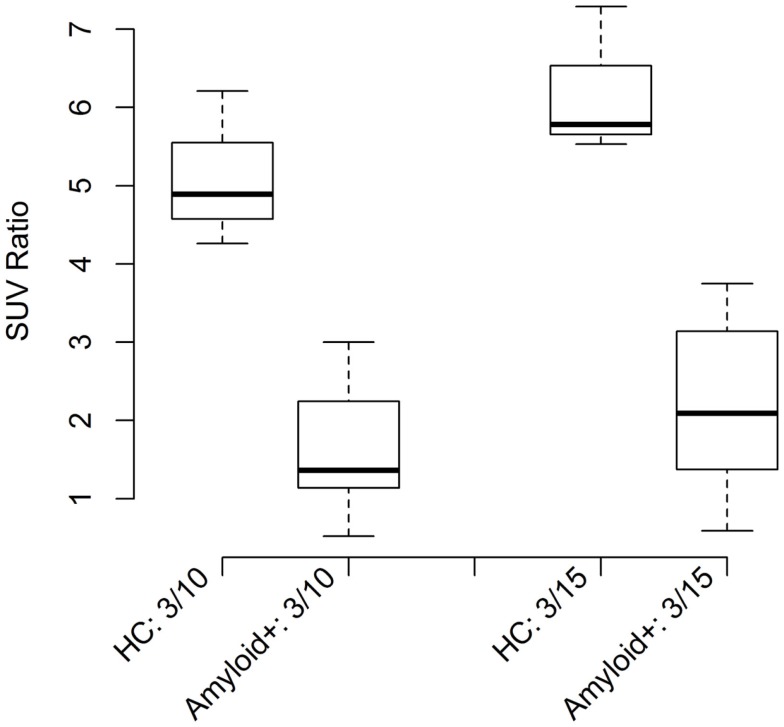
**Shows boxplots of SUV ratios for all healthy and amyloid positive patient groups**.

In addition to DFA, ratio thresholds were assessed using the 95% CI of the mean or mean 2SD method. This simpler, but less rigorous method also resulted in classification of >90% of disease population vs. healthy controls for all ratio models calculated. Boxplots (Figure 3) show the CIs for control and amyloid positive groups further verifying statistical separation using this analysis methodology.

### 3.4 ^18^F-Florbetapir liver analysis

**Figure 4:**
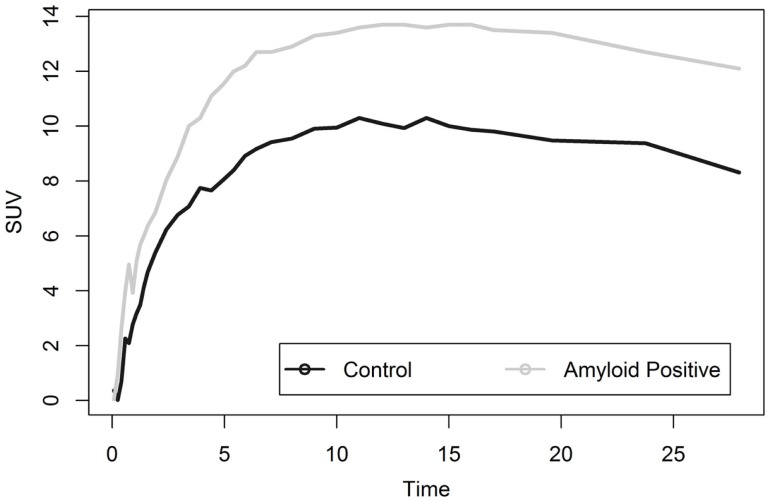
**Shows liver TACs for average values of healthy control and amyloid positive groups**.

**Figure 5:**
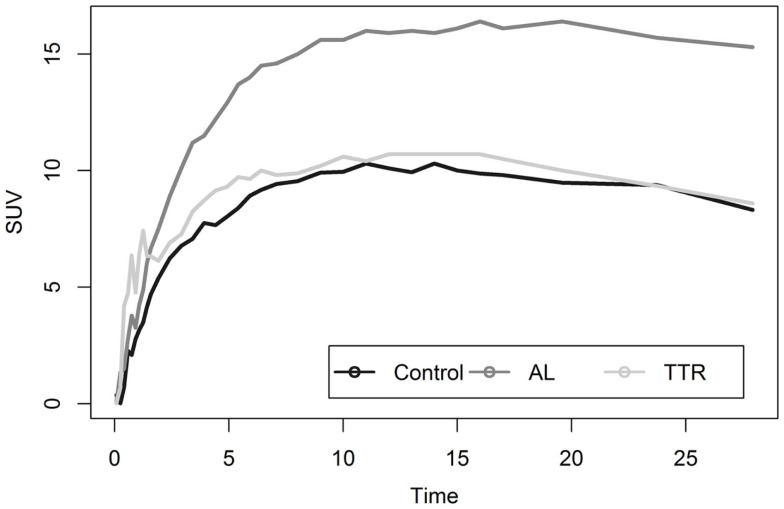
**Shows a comparison of liver uptake in healthy and diseased populations**. These images show that using the liver as a reference point for quantitative measurements is risky as significant uptake is seen even in healthy controls. This is further exacerbated as TAC comparisons between TTR and controls are nearly indistinguishable.

Liver analysis indicated clearly separated TACs for healthy vs. diseased populations when all data were averaged (Figure 4). More detailed analysis of TTR and AL amyloid populations vs. controls showed that TACs for TTR and control groups were very similar while data from AL amyloid patients (Figure 5) were distinctly separated from the other two patient groups. Peak values from TAC measurements were 14.04 for amyloid positive patients and 13.4 for healthy controls. The average difference between any points on the TACs was only 1.52 SUV.

### 3.5 ^82^Rb-Chloride analysis

Quantitative cardiac flow assessments for all patients resulted in rest rates of 1.1–1.7. These results were verified by a board certified radiologist trained in nuclear medicine and cardiac image interpretation to be within normal resting cardiac flow rate ranges. Correlation analysis of flow to patient disease status yielded a non-significant (*p* = 0.742) negative correlation. Assessments of correlation between ratio methods and perfusion results yielded non-significant (*p* ≫ 0.05) negative correlations for all tested methods. These results indicate that no significant correlation was seen between reductions in perfusion performance and uptake of ^18^F-Florbetapir in cardiac amyloid disease.

## 4 Discussion and Conclusion

### 4.1 Study summary and novelty

In this work, we have built upon the foundational work by Dorbala et al. that used ^18^F-Florbetapir to aid in quantitative diagnosis of patients with cardiac amyloidosis. Our primary objective in this study was to develop and assess a clinically relevant quantitative imaging protocol that could be used in routine evaluation of cardiac amyloidosis. We have expanded on previous work by including comparisons of Florbetapir cardiac uptake with clinical perfusion studies and have defined a 20 min study that uses a simple analysis technique. We believe this work provides additional confirmation of the ability of Florbetapir to be used as a tool for diagnosis and assessment of cardiac amyloidosis patients.

### 4.2 Comparison to previous work

#### 4.2.1 Comparison to ^11^C-PiB PET

Previous studies using ^11^C-based compounds are made difficult by a number of factors (19). First is that to access 11C-based compounds are limited only to a very small number of sites preventing widespread proliferation and use of compounds based on that radioisotope. Second, PIB is not available widely for use in the United States without an investigational new drug (IND) application, which also severely hinders its widespread use throughout the North American medical community. Third, and finally, the assessment required to show differences between control and disease populations in this study required dynamic image acquisition of 60-min and complex data modeling and analysis, which is not practical for routine clinical imaging.

#### 4.2.2 Comparison to Other ^18^F-Florbetapir Studies

**Figure 6:**
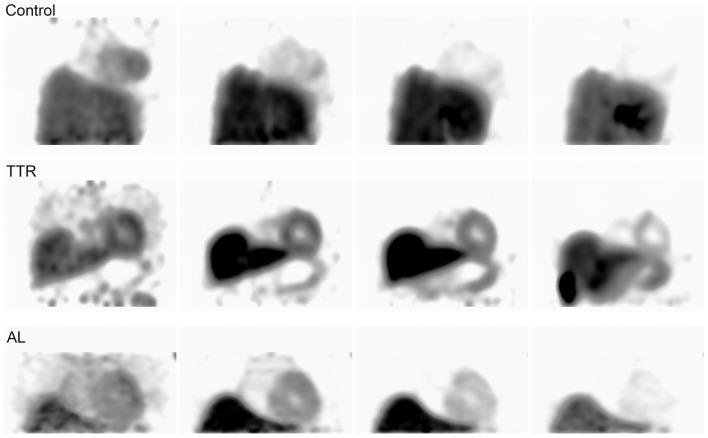
**Shows control and amyloid positive patient data imaged from three of the initial 0–80 min acquisitions**. Each image from left to right shows the progression of time at approximately 5, 10, 30, and 60 min intervals.

Our visual assessments agree with, and confirm, previous cardiac ^18^F-Florbetapir imaging work as we found visual differences between static PET/CT images of HC and patients with MRI-confirmed cardiac amyloidosis. Visual assessments indicated differences between healthy controls and patients with cardiac amyloid involvement in as little as 10 min after injection with only a 10 min acquisition, although even in controls, some background remains in the heart and is never fully cleared even after 80 min of uptake. Visual comparison of controls and disease populations was problematic at later time points. Adding to published results by Dorbala et al. using ^18^F-Florbetapir (21), we found that the ability to perceive and quantify these differences depended heavily on the uptake time prior to acquisition, length of the acquisition, and even window/level settings (Figure 6).

Data from this study also add support to the paradigm that ^18^F-Florbetapir does not necessarily bind avidly to peripheral amyloid but rather is washed-out from amyloid positive tissue at a slower rate. This could be due to low-affinity interactions of the probe with the amyloid fibrils. The transient retention of the probe in amyloid tissue explains why small differences are observed in visual assessments over long dynamic acquisition times. This also potentially explains interesting results from previous whole-body biodistribution studies performed using this compound (29); however, this is an area requiring additional study since this compound was primarily developed for use in Aβ amyloid imaging rather than peripheral AL or TTR amyloid (30).

We have extended our own work in this field as well as that by previous authors (21–23) by creating a clinical protocol that can be completed within 20 min and is easily performed at any PET center. Our proposed analysis enhances previous work by having neither requirement for calculation of ratios between organs nor analysis of blood pool TAC integrals. Our method uses only a single region of interest examined at two time points creating a simple method for analysis that can be used by any trained imaging specialist. We have also provided full guidance on executing this protocol in Section “Appendix” so that others may use this protocol in their practice.

#### 4.2.3 Study Limitations

Although this work has added details regarding quantitative assessment of cardiac amyloid and potential stratification of AL vs. TTR positive patients, we did not focus on any use of this compound in early stage amyloid disease. Additional studies would be required with very specific patient selection in order to determine how adequate this compound is in terms of detecting the presence of amyloid in very early stage patients or within small areas of infiltration.

Our DFA and *t*-test analyses resulted in the statistical separation of all of our samples in this study; however, we have examined only a small sample of patients. With this small population, even with the high degree of statistical significance and power of our studies, it is not possible to conclude that this model would continue to work for a large population of patients. Further trials are needed to assess this model’s ability to diagnose a larger variety of cardiac amyloidosis patients.

This protocol also showed potential for use of ^18^F-Florbetapir imaging in the stratification of AL amyloid from ATTR amyloid using liver and cardiac TACs. Contrary to previous work, our observations from this study indicated statistical separation of data between AL amyloid populations when compared to healthy controls and ATTR amyloid patients, however, ATTR patients were not statistically separated from healthy control. Our contributions show that more data are needed to assess the most appropriate analysis technique for accurate stratification of AL from ATTR using this compound.

Our work is an important addition to the field as it documents a clinically useful 20 min imaging protocol and a simple analysis method for the assessment of cardiac amyloid disease. Development of these simple methods potentially enhances the ability for this compound to be used routinely for assessment of cardiac amyloidosis. More extensive clinical trials would be needed to fully assess the benefit of this compound for identifying patients with cardiac amyloidosis; however, our analysis of PET/CT image data using ^18^F-Florbetapir both confirms previous work in this field and provides important new methods for diagnosing this rare but fatal disease.

## Author Contributions

AS and SA were responsible for injection of Florbetapir and Rubidium imaging agents, imaging, patient record keeping, and manuscript writing. DO and JW were co-PIs on this work responsible for research protocol development, data analysis strategies, and manuscript development. DO was responsible for data and statistical analysis.

## Conflict of Interest Statement

The authors declare that the research was conducted in the absence of any commercial or financial relationships that could be construed as a potential conflict of interest.

## Appendix

### A.1 Clinical protocol

#### A.1.1 Imaging

- Place patient on PET/CT imaging pallet
- Acquire topogram of patient for selection of thoracic scan range
- Perform CT using institutional parameters
- Begin listmode acquisition and wait 5 s
- Inject patient with approximately 10 mCi of ^18^F-Florbetapir
- Perform 20 min PET listmode acquisition
- Histogram data into two 5 min datasets: 0–5, 15–20 min
- Reconstruct histogrammed data using identical parameters.

Additionally, if the clinical viewing software supports visualization of dynamic data, the dataset may be histogrammed using the following parameters:

Frame 1: 0–5 min
Frame 2: 6–14 min
Frame 3: 15–20 min

The corresponding ROI analysis is then only performed on the first and third frames. For non-listmode enabled scanners, the scan may need to be divided into two distinct 5 min acquisitions separated by the appropriate time delay.

- first acquisition: 5 min acquisition beginning at injection
- second acquisition: 5 min acquisition beginning at 15 min p.i.

#### A.1.2 Analysis

- Draw an ROI of at least 1 cc volume in the septal wall on the first time point image
- Copy the ROI to the second time point image.
- Calculate the ratio between the regions of interest.

In both cases, users should check for appropriate registration between the two time points before performing the ROI ratio analysis.

### A.2 Study imaging parameters

This section outlines the imaging parameters for each modality used in this study.

#### A.2.1 CT

- kVp: 120
- mAs: continuously modulated (CareDose, Siemens Medical Solutions USA, Inc.)
- Scan range: thoracic region from upper lung to just below dome of liver
- Contrast: non-contrast enhanced
- Slice thickness: 5 mm slice thickness
- Reconstructed image matrix: 512 × 512
- Pixel size: approximately 1.5 mm.

#### A.2.2 PET

- Acquisition type: listmode
- No. of beds: 1
- Scan time: 20 min
- Injection: approximately 10 mCi ^18^F-Florbetapir
- Reconstructed image matrix: 200 × 200
- Pixel size: approximately 2 mm
- Reconstruction algorithm: OSEM + Resolution Recovery (TRUEX, HD-PET, Siemens Medical Solutions USA, Inc.).
